# Inflammatory bowel diseases and eosinophilic oesophagitis: Two overlapping disorders?

**DOI:** 10.1002/ueg2.12509

**Published:** 2023-12-08

**Authors:** Nathan Grellier, Julien Kirchgesner

**Affiliations:** ^1^ Department of Hepato‐Gastroenterology Poitiers University Hospital Poitiers France; ^2^ Department of Gastroenterology Sorbonne Université INSERM Institut Pierre Louis d’Epidémiologie et de Santé Publique AP‐HP Hôpital Saint‐Antoine Paris France

Inflammatory bowel diseases (IBD) and eosinophilic oesophagitis (EoE) are immune‐mediated disorders of the gastrointestinal (GI) tract with an increasing incidence worldwide.^1^, ^2^ Both diseases are characterised by a loss of tolerance towards luminal antigens favoured by microbiota disruption suggesting that they may share similar pathophysiology.^3^, ^4^ Associations between the two diseases have been suggested in previous studies, especially the risk of developing EoE in individuals with IBD.^5^ However, data regarding the risk of developing IBD in individuals with EoE are scarce.

In the current issue of the United European Gastroenterology Journal, Uchida et al. assessed the risk of developing IBD in individuals with histopathologic‐proven diagnosis of EoE.^6^ Between 1990 and 2017, using a Swedish nationwide histopathologic database, the authors identified 1587 individuals with EoE. Each of them was matched to five general population reference individuals according to age, sex, and year of diagnosis of EoE, with a total of 7808 individuals. Individuals with EoE were excluded if they had a prior diagnosis of IBD and the follow‐up ended in December 2019. Of note, individuals with EoE underwent more endoscopies during follow‐up than the reference individuals. Overall, 16 EoE individuals (0.01%) and 21 reference individuals (0.003%), were diagnosed with IBD during follow‐up. The risk of developing IBD was 3.5‐fold higher in the EoE group compared to matched individuals from the general population. In subgroup analyses, individuals with EoE were at higher risk of developing later Crohn's disease (CD), but not ulcerative colitis (UC). These results suggest that only CD could be associated with EoE. Additionally, the risk of being diagnosed with EoE was 15 times higher in patients with IBD compared to the general population, suggesting a bidirectional association. Some limitations need to be acknowledged. First, surveillance bias could occur as individuals with EoE were more likely to have upper GI endoscopies during follow‐up that could identify lesions related to CD. Patients with EoE are more prone to being followed by GI doctors and so undergo subsequent GI endoscopies. Second, important confounding factors are not collected in the Swedish administrative healthcare databases, notably smoking or medications such as proton pump inhibitors, and residual confounding cannot be excluded.

The study by Uchida et al. provides important insights on the occurrence of these two diseases. These results confirm the association of IBD and EoE already described in a study based on U.S insurance claims data.^5^ In this study, the risk of EoE was higher in CD but also in UC, while the risk of IBD was higher among patients with EoE. Patients with both diseases appeared to have more IBD‐related complications compared to patients only diagnosed with IBD. Only few prospective cohort studies described the clinical characteristics and treatment outcomes of concomitant EoE and IBD.^7^ The relatively low incidence of the two concomitantly diagnoses makes it difficult to draw conclusions about the bidirectional impact of the two diseases.

Eosinophils reside in the lamina propria of the GI tract and play an important role in the immune response as a defensive role against viral and parasitic pathogens. The association between IBD and increased levels of circulating and mucosal eosinophils is well established, but its impact on IBD disease natural history and severity is still debated.^8^, ^9^ Recent study from in vitro colitis models, showed the important role of active eosinophils in interleukine‐33 and interferon‐gamma pathways.^10^, ^11^ Using human tissue, active eosinophils were more present in patients with IBD compared to healthy subjects especially in the lumen pole of the epithelial barrier suggesting that their presence could drive local inflammation. Yet, the role of eosinophils in intestinal inflammation remains to be explored. Targeting eosinophils with biologic therapies using dupilumab, a biologic targeting interleukine‐4 and interleukine‐33, have already been seen in EoE and other diseases such as chronic obstructive pulmonary disease (COPD).^12^, ^13^ In EoE, dupilumab was effective in the overall population of patients with EoE, while dupilumab interestingly only proved its efficacy for the treatment of COPD in patients with peripheral eosinophilia. One phase 2 trial assessing the efficacy and safety of dupilumab therapy in patients with UC with an eosinophilic phenotype is currently in the recruitment phase.^14^

In conclusion, the findings from Uchida et al. suggest that immune dysregulation caused by EoE may lead to a susceptibility to develop IBD. Based upon this and other studies, patients diagnosed with both conditions may activate specific inflammatory pathways that include eosinophils with potential therapeutic implications in IBD.

## Data Availability

No data are shared in this editorial.
